# An anti-CD6 antibody for the treatment of COVID-19 patients with cytokine-release syndrome: report of three cases

**DOI:** 10.2217/imt-2020-0235

**Published:** 2021-01-05

**Authors:** Lázaro Manuel Filgueira, Julio Betancourt Cervantes, Orlando Adolfo Lovelle, Carlos Herrera, Carlos Figueredo, Jorge Alain Caballero, Naivy Sánchez, Jorge Berrio, Geidy Lorenzo, Meylan Cepeda, Mayra Ramos, Danay Saavedra, Ana Laura Añe-Kouri, Zaima Mazorra, Kalet Leon, Tania Crombet, Armando Caballero

**Affiliations:** ^1^Manuel Piti Fajardo University Hospital, Ciudad Escolar Abel Santamaría. U/M 9958, Santa Clara city, Villa Clara, Cuba; ^2^Arnaldo Milián University Hospital, Santa Clara St., Santa Clara city, Villa Clara, Cuba; ^3^Center of Molecular Immunology, 216 St, corner 15, Atabey, Havana, Cuba; ^4^Superior Institute of Basic & Preclinical Sciences of Havana “Victoria de Girón”, Street 25, Playa, Havana, Cuba

**Keywords:** CD6 molecule, coronavirus 2, COVID-19, cytokine-release syndrome, IL-6, itolizumab, monoclonal antibody, SARS-CoV-2

## Abstract

In COVID-19, the inflammatory cytokine-release syndrome is associated with the progression of the disease. Itolizumab is a monoclonal antibody that recognizes human CD6 expressed in activated T cells. The antibody has shown to be safe and efficacious in the treatment of moderate to severe psoriasis. Its effect is associated with the reduction of pro-inflammatory cytokines release, including IFN-γ, IL-6 and TNF-α. Here, we report the outcome of three severe and critically ill COVID-19 patients treated with itolizumab as part of an expanded access protocol. Itolizumab was able to reduce IL-6 concentrations in all the patients. Two of the three patients showed respiratory and radiological improvement and were fully recovered. We hypothesize this anti-inflammatory therapy in addition to antiviral and anticoagulant therapy could reduce COVID-19 associated morbidity and mortality.

## Lay abstract

In COVID-19 patients, inflammation is associated with the progression of the disease. This inflammation comes from immune system activation in response to SARS-COV-2. Itolizumab is an antibody that recognizes a protein expressed in immune cells. The antibody has shown to be safe and efficacious in the treatment of moderate to severe psoriasis. Its effect is associated with the reduction of inflammation and immune system activation. Here, we report the outcome of three severe and critically ill COVID-19 patients treated with itolizumab as part of an expanded access clinical protocol. Itolizumab was able to reduce inflammation in all patients. Two of the three patients showed respiratory and radiological improvement and were fully recovered. We hypothesize this anti-inflammatory therapy in addition to antiviral and anticoagulant therapy could reduce COVID-19 associated morbidity and mortality.

A group of COVID-19 patients develops severe or critical disease accompanied by the cytokine storm syndrome. Cytokines are essential to the pathophysiology of disease and IL-6, IL-1 and TNF-α appear to be harmful, particularly in the context of the cytokine-release syndrome (CRS) [1,2].

CD6 is a membrane glycoprotein expressed primarily in mature, activated T cells. Ligand binding of CD6, increases events such as adhesion, activation, proliferation, differentiation and survival [3–6]. In addition, CD6 mediates interaction between T cells and antigen-presenting cells, contributing to the maturation of immune synapses [6]. CD6-mediated costimulation contributes to the maturation of a Th1 pattern in human T cells and preferentially promotes a pro-inflammatory response characterized by the secretion of TNF-α, IL-6 and IFN γ [7].

The activated leukocyte-cell adhesion molecule (ALCAM), also known as CD166, has been identified as the CD6 ligand [8,9]. ALCAM interaction with CD6 stabilizes the formation of the immune synapse between the lymphocytes and the antigen-presenting cells [5].

Itolizumab is a humanized monoclonal antibody developed at the Center of Molecular Immunology, in Cuba, that recognizes a region in the distal domain of the human CD6 [10,11]. The antibody reduces the expression of the intracellular proteins involved in activation and inhibits the proliferation of T cells, even in the presence of the ALCAM and IL-2 [10–12]. The effect is associated with the reduction of the secretion of pro-inflammatory cytokines including IFN-γ, IL-6 and TNF-α [13,14]. The antibody has demonstrated to be safe and efficacious in patients with moderate to severe psoriasis [15,16]. Studies from blood and tissue samples from patients with severe psoriasis showed that itolizumab reduces the proliferation of T cells and serum concentration of IL-6, TNF-α and IFN-γ [13–16]. A significant reduction of the inflammatory pattern was also observed in the tissue. On account of its well proven effect on T-cell activation and proinflammatory cytokines production, itolizumab was included as part of a Cuban expanded access protocol in critical, severe and moderate COVID-19 patients with high risk of aggravation. The study will be published soon (manuscript accepted) and here we describe in detail the outcome of three initial patients receiving the antibody.

## Cases description

**Figure 1.**
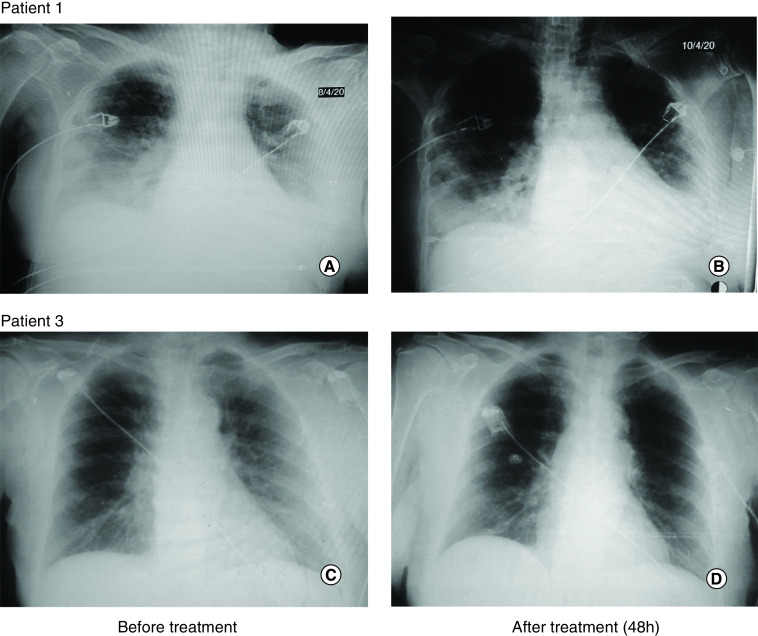
Radiological images before and after one dose of itolizumab. **Patient 1** **(A)** Before itolizumab (D0): diffuse Interstitial-alveolar infiltrate in both lung fields, predominantly in the bases. Minor left pleural effusion. **(B)** After itolizumab (48 h): radiological improvement with decreased diffuse infiltrate and radio-opacity at the top of both lungs and in both lung bases, in less proportion. The minor pleural effusion persists. **Patient 3** **(C)** Before itolizumab (D0): alveolar interstitial inflammatory infiltrate in both lung fields, predominantly on the right side. **(D)** After itolizumab (48 h): radiological improvement with significant decrease of the alveolar interstitial infiltrate of both lungs.

**Figure 2.**
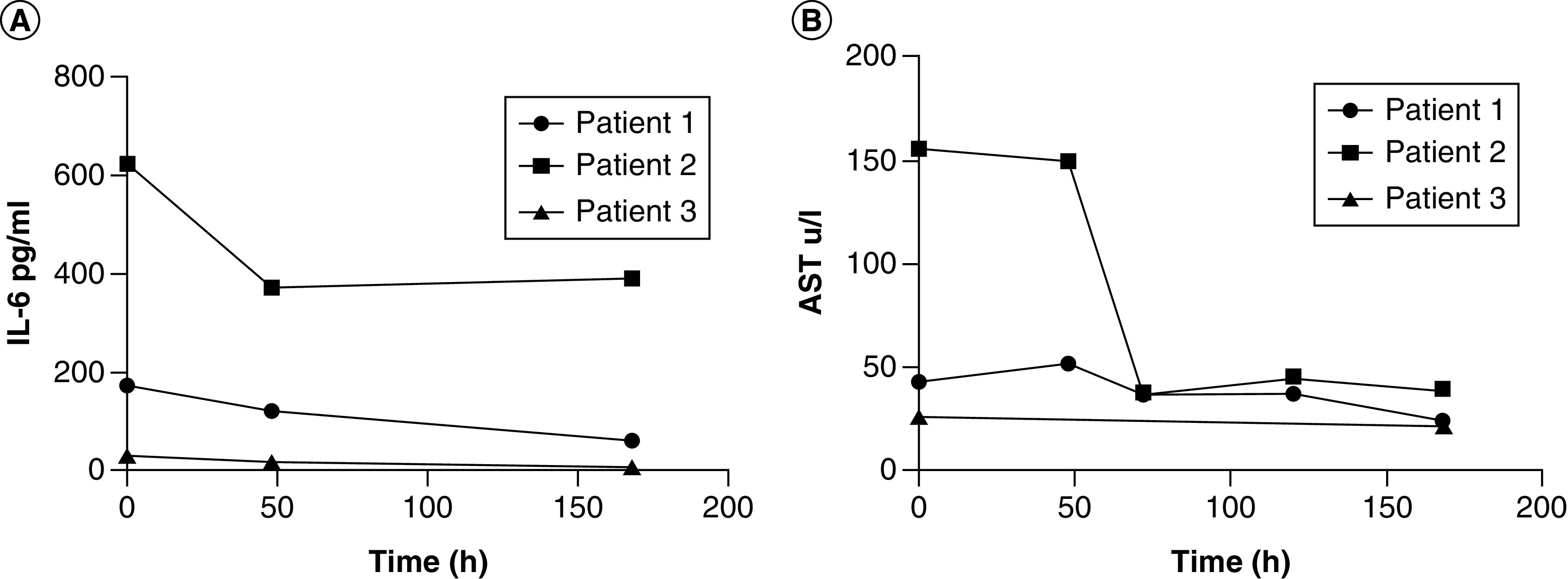
Circulating biomarkers in patient's sera. IL-6 and AST concentrations kinetics **(A & B, respectively)** measured in patients during itolizumab treatment.

**Patient 1**: A 53-year old woman with a personal history of essential hypertension and Type 2 diabetes mellitus presented with symptoms of polypnea of more than 40 rpm, use of respiratory ancillary musculature and dry cough. She arrived from a foreign country where COVID-19 was spreading. Symptoms started on 23 March and COVID-19 was diagnosed on 26 March on the basis of positive real time PCR (RT-PCR) for SARS-CoV-2. Initial gasometry showed moderate hypoxemia and respiratory alkalosis with a partial pressure of oxygen/fraction of inspired oxygen (PO_2_/FiO_2_) ratio of 191. Chest x-rays showed interstitial lesions in both lung fields. She started therapy with lopinavir/ritonavir, chloroquine, recombinant IFN α-2b and rocephin. In spite of treatment, the illness subsequently progressed to hypoxemic respiratory failure warranting the initiation of invasive mechanical ventilation. At day 13 of her admission in the ICU, she showed radiologic worsening of the interstitial multifocal pneumonia, with elevation of ALP, LDH, erythrocyte sedimentation rate and D-dimer. Physicians administered itolizumab at a dose of 200 mg. After 48 h of the first itolizumab dose, PO2/FiO2 improved and there were evidences of radiological improvement (Figure 1A & B). Patient was extubated after the first dose of the antibody and her status changed from critical to severe. She received a second dose of the antibody (200 mg), 48 h after the first infusion. 3 days after the first administration, patient was hemodynamically stable and has spontaneous ventilation. IL-6 levels were evaluated before itolizumab administration and after 2 and 7 days of the first administration. IL-6 levels reduced overtime from 172 pg/ml to 60 pg/ml (day 7) as depicted in Figure 2A. IL-1 was evaluated at the same time intervals, but it was undetectable. In addition, aspartate amino transferase (AST) concentrations were evaluated at different time points showing a reduction from 43 U/l to 24 U/l after 7 days (Figure 2B). No adverse events related with itolizumab were reported.

**Patient 2**: An 89-year old man with a personal history of chronic ischemic cardiopathy, permanent atrial fibrillation, hypertension and hypothyroidism. He has a previous history of alcoholism and several hospital admissions in the last 3 months on account of infectious respiratory diseases. He came to the hospital on 2 April after 7 days of shortness of breath, fever, asthenia and dry cough. He was in contact with a person who returned from a foreign country, where COVID-19 was extending. Physical examination shows signs of respiratory failure characterized by tachycardia, polypnea, intercostal and supraclavicular muscle retraction, high blood pressure, oxygen saturation of 82%, poor diuresis and drowsiness. Chest x-rays showed bilateral pulmonary inflammatory infiltrates, predominantly in the right lung. Admission ECG showed an atrial fibrillation with rapid ventricular response and the initial gasometry showed severe hypoxemia and respiratory alkalosis. Patient also had leukocytosis, altered globular sedimentation rate as well as elevated values of AST, LDH, D-dimer and positive C-reactive protein. He was admitted into the ICU requiring invasive mechanical ventilation. Treatment with lopinavir–ritonavir, chloroquine, IFN α-2b, meropenem and linezolid was initiated. 3 days after his admission into the ICU, itolizumab was prescribed, due to worsening of the bilateral pulmonary infiltrates together with a deterioration of the ventilatory function (PO2/FiO2 = 173). After the first antibody infusion, PO2/FiO2 significantly increased (PO2/FiO2 = 320) and there were evidences of radiological improvement. 3 days after, patient showed radiological worsening of the left lung, characterized by alveolar hypoventilation and atelectasis; then, a 70% pneumothorax was established. Treatment of the pneumothorax with minimal pleurotomy was very demanding and required 3 days for the resolution. The patient received a second infusion of the monoclonal antibody 72 h after the first, while a third dose was administered at the discretion of the treating physicians, 2 days after the second. In total, patient received three doses of itolizumab (200 mg) without any related adverse event. IL-6 levels were evaluated before itolizumab administration and after 2, 4 and 7 days of the first administration. IL-6 was extremely high at baseline (623 pg/ml) and even though cytokine levels reduced roughly 50%, the lowest value remained above 300 pg/ml after 7 days. IL-6 kinetics is shown in Figure 2A. Apart from IL-6, IL-1 was undetectable at this time point of the disease. Interesting, in the case of AST, a significant reduction is detecting at day 7 (pretreatment: 156 U/l, D7: 40 U/l) as is showed in Figure 2B. After 10 days of admission into the ICU, patient presented a myocardial dysfunction and shock that required vasoactive support with norepinephrine. On day 13, he finally died, on account of a mixed cardiovascular and respiratory failure.

**Patient 3**: An 81-year old female, who is COVID-19 positive contact was not identified at the moment of hospitalization. She started symptoms on 2 April and entered the ICU on 5 April. Patient has a previous history of hypertension, diabetes mellitus, glaucoma and smoking habit. She was admitted with frequent cough, wheezing and diarrhea. The diagnosis of viral pneumonia by COVID-19 was confirmed by RT-PCR on 7 April. Oxygen support at 5 l/min and treatment with ceftriaxone, lopinavir–ritonavir, chloroquine and IFN α-2b was indicated. Chest images showed bilateral interstitial infiltration in both lungs. Patient condition was classified as severe, although she did not require invasive mechanical ventilation. A dose of itolizumab (200 mg) was given the day after her admission into the ICU. Two days after the antibody administration, together with the rest of the therapy, there was an improvement of the respiratory distress while the chest image showed a decrease of the alveolar interstitial infiltrate of both lungs (Figure 1C & D). Patient left the ICU after a favorable clinical and radiological evolution. IL-6 concentration was measured prior and after the antibody administration. IL-6 level at baseline was lower than in previous cases (30 pg/ml), but also decreased 48 h and 168 h after itolizumab infusion, in parallel with patient recovery (Figure 2A). IL-1 and TNF-α were untraceable before and 48 h after the antibody administration. Regarding to AST concentration, the values were in the normal range during the treatment period (Figure 2B).

## Materials & methods

Three patients with diagnosis of COVID-19 classified as critically ill (two) and severely ill (one) were included in an expanded access trial to receive itolizumab in addition to standard treatment (http://rpcec.sld.cu/trials/RPCEC00000311-En). The study was approved by a central ethical review board, created especially for COVID-19 and by the Cuban Regulatory Agency (CECMED). Before the treatment, informed consent was obtained from enrolled patient.

Laboratory results included blood routine, leucocyte subsets and blood biochemical parameters were collected. The level of inflammatory cytokines was measured using human validated commercially available kit from R&D Systems (MN, USA): human IL-6 Quantikine ELISA Kit (Cat# S6050), human IL-1 beta/IL-1F2 Quantikine ELISA Kit (Cat# SLB50) and human TNF-α Quantikine ELISA Kit (Cat# STA00D). In the case of IL-6, the normal range of this cytokine in sera is between 0 and 7 pg/ml [17]. We used intra-assay precision and inter-assay precision methods to evaluate precision in this study.

For intra-assay precision (precision within an assay), three samples of known concentration were tested on one plate to assess intra-assay precision.

For inter-assay precision (precision between assays), three samples of known concentration were tested in separate assays to assess inter-assay precision.

## Discussion

CRS is characterized by high levels of inflammatory cytokines. Among them, IL-6 is considered a major mediator of this hyperinflammation [18,19]. Most of the COVID-19 patients-related papers showed the CRS as one of the main hallmark of the disease [20]. Additionally, IL-6 levels predict severity in COVID-19 patients [21]. There is high heterogeneity of IL-6 values among papers for classifying severe and nonsevere patients. In a large meta-analysis of 52 manuscripts, Elshazli and coworkers found that the IL-6 level associated with COVID-19 severity was 22.9 pg/ml [22]. In a series of patients treated with itolizumab a cutoff of 28 pg/ml discriminates severe patients from nonsevere (manuscript just accepted in Immunity and Aging). In the present study, the IL-6 concentration of the three patients was above this cutoff. It confirms the classification of these patients as severe and that the CRS is probably occurring.

Several anticytokine therapies have been tried for treating the hyperinflammatory phase of COVID-19 [20,23–25]. Here, we report the use of a well-known anti-inflammatory antibody targeting CD6 to treat the CRS arising in COVID-19 patients. Using an anti-CD6 antibody could reduce the concentration of several pro-inflammatory cytokines, including IL-6, IFN-γ, TNF-α and IL-17, among others, representing an advantage as compared with single-cytokine targeting antibodies. The antibody would not exacerbate lymphopenia since it does not induce complement or antibody dependent cytotoxicity [10,11].

These three SARS-CoV2 patients developed severe respiratory distress together with multifocal interstitial pneumonia. Two patients required invasive mechanical ventilation while the third patient only needed oxygen supply. They all received the anti-CD6 antibody itolizumab in combination with other drugs incorporated into the Cuban national protocol, including lopinavir/ritonavir, chloroquine and IFNα-2b. Regarding to laboratory parameters, AST is strongly associated with mortality risk compared with other parameters, reflecting liver and kidney injury [26,27]. In our cases, critically ill patients showed a significant reduction of AST concentration after the treatment suggesting an improvement in the function of these organs.

Itolizumab was very safe and did not seem to exacerbate opportunistic secondary infections. Unfortunately, we did no measure neither the frequency nor the total amount of circulating T cells in these initial patients. The impact of itolizumab on circulating T cells are being implementing in new recruited patients.

Our preliminary findings support that IL-6 levels correlated with the severity of the disease and that the antibody was capable of reducing IL-6 concentration in all three subjects. One patient died after subsequent respiratory and cardiovascular complications. In this particular case, IL-6 concentration was extremely elevated at the moment of itolizumab infusion. Although, the levels of IL-6 decreased during treatment, the values at 7 days kept still very high (above 300 pg/ml). There was a transient improvement of respiratory function but it was not enough. Presumably, the consequences of the hyperinflammatory syndrome (thrombosis, alveolar damage and severe tissue hypoxia) were irreversible at the moment of treatment and patient died as consequence of these factors.

## Conclusion

In summary, in these severe and critically ill patients, itolizumab was able to reduce IL-6 concentrations. Notably, itolizumab-related adverse events were not reported. Patients with baseline IL-6 levels below 200 pg/ml, showed prompt clinical and radiological recovery. We anticipate that the timely use of this anti-inflammatory antibody in combination with the appropriate antiviral and anticoagulant therapy could reduce the mortality associated with COVID-19. The analysis of the complete-series will be published shortly.

**Summary points**

- Inflammatory cytokine-release syndrome is associated with the progression of the coronavirus disease (COVID-19).
- No current therapy has proven effective for the management of this syndrome so far.
- Itolizumab is a humanized monoclonal antibody that recognizes human CD6 and its effect is associated with the reduction of pro-inflammatory cytokines release.
- We present three COVID-19 cases who developed severe respiratory distress together with multifocal interstitial pneumonia.
- The patients were treated with itolizumab combined with antiviral therapies.
- Itolizumab reduced circulating IL-6 concentrations in the three COVID-19 patients.
- Two patients showed rapid ventilatory and radiological improvement and were fully recovered.
- Itolizumab-related adverse events were not reported.
- These cases show that the timely use of this anti-inflammatory antibody in combination with the appropriate antiviral and anticoagulant therapy could reduce the mortality associated with COVID-19.

## References

[1] Jamilloux Y, Henry T, Belot A Should we stimulate or suppress immune responses in COVID-19? Cytokine and anti-cytokine interventions. Autoimmun. Rev. 19(7), 102567 (2020). 32376392 10.1016/j.autrev.2020.102567 PMC7196557

[2] Chakraborty C, Sharma AR, Sharma G, Bhattacharya M, Lee SS SARS-CoV-2 causing pneumonia-associated respiratory disorder (COVID-19): diagnostic and proposed therapeutic options. Eur. Rev. Med. Pharmacol. Sci. 24(7), 4016–4026 (2020). 32329877 10.26355/eurrev_202004_20871

[3] Singer NG, Fox DA, Haqqi TM CD6: expression during development, apoptosis and selection of human and mouse thymocytes. Int. Immunol. 14(6), 585–597 (2002). 12039910 10.1093/intimm/dxf025

[4] Gimferrer I, Calvo M, Mittelbrunn M Relevance of CD6-mediated interactions in T cell activation and proliferation. J. Immunol. 173(4), 2262–2270 (2004). 15294938 10.4049/jimmunol.173.4.2262

[5] Zimmerman AW, Joosten B, Torensma R, Parnes JR, van Leeuwen FN, Figdor CG Long-term engagement of CD6 and ALCAM is essential for T-cell proliferation induced by dendritic cells. Blood 107(8), 3212–3220 (2006). 16352806 10.1182/blood-2005-09-3881

[6] Ibanez A, Sarrias MR, Farnos M Mitogen-activated protein kinase pathway activation by the CD6 lymphocyte surface receptor. J. Immunol. 177(2), 1152–1159 (2006). 16818773 10.4049/jimmunol.177.2.1152

[7] Nair P, Melarkode R, Rajkumar D, Montero E CD6 synergistic co-stimulation promoting proinflammatory response is modulated without interfering with the activated leucocyte cell adhesion molecule interaction. Clin. Exp. Immunol. 162(1), 116–130 (2010). 20726988 10.1111/j.1365-2249.2010.04235.x PMC2990937

[8] Bodian DL, Skonier JE, Bowen MA Identification of residues in CD6 which are critical for ligand binding. Biochemistry 36(9), 2637–2641 (1997). 9054570 10.1021/bi962560+

[9] Whitney GS, Starling GC, Bowen MA, Modrell B, Siadak AW, Aruffo A The membrane-proximal scavenger receptor cysteine-rich domain of CD6 contains the activated leukocyte cell adhesion molecule binding site. J. Biol. Chem. 270(31), 18187–18190 (1995). 7543097 10.1074/jbc.270.31.18187

[10] Rodriguez PC, Torres-Moya R, Reyes G A clinical exploratory study with itolizumab, an anti-CD6 monoclonal antibody, in patients with rheumatoid arthritis. Results Immunol. 2, 204–211 (2012). 24371585 10.1016/j.rinim.2012.11.001 PMC3862386

[11] Hernandez P, Moreno E, Aira LE, Rodriguez PC Therapeutic targeting of CD6 in autoimmune diseases: a review of cuban clinical studies with the antibodies IOR-T1 and itolizumab. Curr. Drug Targets 17(6), 666–677 (2016). 26844560 10.2174/1389450117666160201114308

[12] Dogra S, Uprety S, Suresh SH Itolizumab, a novel anti-CD6 monoclonal antibody: a safe and efficacious biologic agent for management of psoriasis. Expert Opin. Biol. Ther. 17(3), 395–402 (2017). 28064543 10.1080/14712598.2017.1279601

[13] Aira LE, Hernandez P, Prada D Immunological evaluation of rheumatoid arthritis patients treated with itolizumab. MAbs 8(1), 187–195 (2016). 26466969 10.1080/19420862.2015.1105416 PMC4966522

[14] Aira LE, Lopez-Requena A, Fuentes D Immunological and histological evaluation of clinical samples from psoriasis patients treated with anti-CD6 itolizumab. MAbs 6(3), 783–793 (2014). 24594862 10.4161/mabs.28376 PMC4011922

[15] Krupashankar DS, Dogra S, Kura M Efficacy and safety of itolizumab, a novel anti-CD6 monoclonal antibody, in patients with moderate to severe chronic plaque psoriasis: results of a double-blind, randomized, placebo-controlled, Phase-III study. J. Am. Acad. Dermatol. 71(3), 484–492 (2014). 24703722 10.1016/j.jaad.2014.01.897

[16] Dogra S, SK D, Budamakuntla L Long-term efficacy and safety of itolizumab in patients with moderate-to-severe chronic plaque psoriasis: a double-blind, randomized-withdrawal, placebo-controlled study. J. Am. Acad. Dermatol. 73(2), 331–333 e331 (2015). 26183983 10.1016/j.jaad.2015.03.040

[17] Xu X, Han M, Li T Effective treatment of severe COVID-19 patients with tocilizumab. Proc. Natl Acad. Sci. USA 117(20), 10970–10975 (2020). 32350134 10.1073/pnas.2005615117 PMC7245089

[18] McGonagle D, Sharif K, O'Regan A, Bridgewood C The role of cytokines including interleukin-6 in COVID-19 induced pneumonia and macrophage activation syndrome-like disease. Autoimmun. Rev. 19(6), 102537 (2020). 32251717 10.1016/j.autrev.2020.102537 PMC7195002

[19] Giamarellos-Bourboulis EJ, Netea MG, Rovina N Complex immune dysregulation in COVID-19 patients with severe respiratory failure. Cell Host Microbe. 27(6), 992–1000 e1003 (2020). 32320677 10.1016/j.chom.2020.04.009 PMC7172841

[20] Saha A, Sharma AR, Bhattacharya M, Sharma G, Lee SS, Chakraborty C Tocilizumab: a therapeutic option for the treatment of cytokine storm syndrome in COVID-19. Arch. Med. Res. 51(6), 595–597 (2020). 32482373 10.1016/j.arcmed.2020.05.009 PMC7241374

[21] Chen X, Zhao B, Qu Y Detectable serum severe acute respiratory syndrome coronavirus 2 viral load (RNAemia) is closely correlated with drastically elevated interleukin 6 level in critically ill patients with coronavirus disease 2019. Clin. Infect. Dis. 71(8), 1937–1942 (2020). 32301997 10.1093/cid/ciaa449 PMC7184354

[22] Elshazli RM, Toraih EA, Elgaml A Diagnostic and prognostic value of hematological and immunological markers in COVID-19 infection: a meta-analysis of 6320 patients. PLoS ONE 15(8), e0238160 (2020). 32822430 10.1371/journal.pone.0238160 PMC7446892

[23] Mehta P, McAuley DF, Brown M COVID-19: consider cytokine storm syndromes and immunosuppression. Lancet 395(10229), 1033–1034 (2020). 32192578 10.1016/S0140-6736(20)30628-0 PMC7270045

[24] Calabrese C, Rajendram P, Sacha G, Calabrese L Practical aspects of targeting IL-6 in COVID-19 disease. Cleve. Clin. J. Med. (2020) (Epub ahead of print). 10.3949/ccjm.87a.ccc018 32409439

[25] Chakraborty C, Sharma AR, Bhattacharya M, Sharma G, Lee SS, Agoramoorthy G COVID-19: consider IL-6 receptor antagonist for the therapy of cytokine storm syndrome in SARS-CoV-2 infected patients. J. Med. Virol. 92(11), 2260–2262 (2020). 32462717 10.1002/jmv.26078 PMC7283789

[26] Lei F, Liu YM, Zhou F Longitudinal association between markers of liver injury and mortality in COVID-19 in China. Hepatology 72(2), 389–398 (2020). 32359177 10.1002/hep.31301 PMC7267515

[27] Henry BM, de Oliveira MHS, Benoit S, Plebani M, Lippi G Hematologic, biochemical and immune biomarker abnormalities associated with severe illness and mortality in coronavirus disease 2019 (COVID-19): a meta-analysis. Clin. Chem. Lab. Med. 58(7), 1021–1028 (2020). 32286245 10.1515/cclm-2020-0369

